# Molecular Characterization of Circulating Tumor Cells Enriched by A Microfluidic Platform in Patients with Small-Cell Lung Cancer

**DOI:** 10.3390/cells8080880

**Published:** 2019-08-13

**Authors:** Eva Obermayr, Christiane Agreiter, Eva Schuster, Hannah Fabikan, Christoph Weinlinger, Katarina Baluchova, Gerhard Hamilton, Maximilian Hochmair, Robert Zeillinger

**Affiliations:** 1 Molecular Oncology Group, Department of Obstetrics and Gynecology, Comprehensive Cancer Center, Medical University of Vienna, Waehringer Guertel 18-20, 1090 Vienna, Austria; 2 Department of Respiratory and Critical Care Medicine, Sozialmedizinisches Zentrum Baumgartner Höhe, Sanatoriumstrasse 2, 1140 Vienna, Austria; 3 Division of Oncology, Biomedical Center Martin, Jessenius Faculty of Medicine in Martin, Comenius University in Bratislava, Malá Hora 4C, 036 01 Martin, Slovakia; 4 Department of Surgery, Medical University of Vienna, Waehringer Guertel 18-20, 1090 Vienna, Austria

**Keywords:** small-cell lung carcinoma, circulating tumor cells, microfluidics, gene expression analysis, synaptophysin, chromogranin A, rovalpituzumab tesirine

## Abstract

At initial diagnosis, most patients with small-cell lung cancer (SCLC) present with metastatic disease with a high number of tumor cells (CTCs) circulating in the blood. We analyzed RNA transcripts specific for neuroendocrine and for epithelial cell lineages, and Notch pathway delta-like 3 ligand (*DLL3*), the actionable target of rovalpituzumab tesirine (Rova-T) in CTC samples. Peripheral blood samples from 48 SCLC patients were processed using the microfluidic Parsortix™ technology to enrich the CTCs. Blood samples from 26 healthy donors processed in the same way served as negative controls. The isolated cells were analyzed for the presence of above-mentioned transcripts using quantitative PCR. In total, 16/51 (31.4%) samples were CTC-positive as determined by the expression of epithelial cell adhesion molecule 1 (*EpCAM)*, cytokeratin 19 (*CK19)*, chromogranin A (*CHGA)*, and/or synaptophysis (*SYP)*. The epithelial cell lineage-specific *EpCAM* and/or *CK19* gene expression was observed in 11 (21.6%) samples, and positivity was not associated with impaired survival. The neuroendocrine cell lineage-specific *CHGA* and/or *SYP* were positive in 13 (25.5%) samples, and positivity was associated with poor overall survival. *DLL3* transcripts were observed in four (7.8%) SCLC blood samples and *DLL3*-positivity was similarly associated with poor overall survival (OS). CTCs in SCLC patients can be assessed using epithelial and neuroendocrine cell lineage markers at the molecular level. Thus, the implementation of liquid biopsy may improve the management of lung cancer patients, in terms of a faster diagnosis, patient stratification, and on-treatment therapy monitoring.

## 1. Introduction

Lung cancer is the most common cancer worldwide. In 2018, a total of 2.1 million new cases were estimated, accounting for 11.6% of all new cancer diagnoses [1,2]. In general, two major types of lung cancer exist: non-small-cell lung cancer (NSCLC), which accounts for about 85% of all lung cancer cases, and small-cell lung cancer (SCLC), which is diagnosed in approximately 15% of all lung cancers. For patients with early-stage NSCLC, a surgical resection offers the best opportunity for cure, while in advanced cases a systemic therapy is the standard of care. SCLC, however, is usually diagnosed rather late when the cancer has already disseminated. In this case a multimodal therapy which includes chemotherapy and radiotherapy is considered the gold standard [3]. Due to these different therapeutic approaches, it is of utmost importance to have a reliable diagnostic platform to differentiate between SCLC and NSCLC.

SCLC belongs to the group of neuroendocrine tumors of the lung. It is diagnosed using hematoxylin and eosin stained sections of the biopsied tissue. However, the histopathological diagnosis of SCLC based on its distinctive morphology may be difficult due to limited material supply from biopsied tissue or aspirated cytological specimens [4]. In some cases the diagnosis of SCLC may be further confirmed by immunohistochemistry using the neuroendocrine markers chromogranin (CHGA), synaptophysin (SYP), and neural cell adhesion molecule 1 (NCAM1) [5,6]. In recent years, the Notch pathway delta-like 3 ligand (DLL3) has gradually gained more interest since it is frequently and selectively expressed on tumorous tissue in SCLC patients and hence it has been associated with neuroendocrine tumorigenesis. Most importantly it is the therapeutic target of the antibody-drug conjugate rovalpituzumab tesirine (Rova-T) [7].

In contrast to conventional tissue biopsies or cytological preparations, liquid biopsies that contain circulating tumor cells (CTCs) and/or circulating tumor DNA, represent a novel approach that illuminates the whole molecular profile of a tumor at the time of sampling [8,9]. Liquid biopsies are taken by a simple blood draw and, thus, are less stressful for the patient, more conventionally used and less expensive than tissue biopsies. For this reason, liquid biopsies can be taken several times to monitor the temporal heterogeneity of the tumor. Especially in lung cancer, liquid biopsies may outperform tissue biopsies with respect to the tumor’s accessibility at resection. In addition, small tissue samples are often already exhausted after routine histological staining and hence no longer available for advanced analysis. Furthermore, longitudinal sampling for monitoring of any development of therapy resistance is almost impossible with tissue biopsies [10].

The presence and clinical significance of CTCs has already been shown in many types of malignancies, among them e.g., breast, colorectal, prostate, and lung cancer. In contrast to most other cancer types, SCLC is characterized by a large number of CTCs in the circulation [11]. Several studies have shown the prognostic value of CTC counts in SCLC, most of them using the US Food and Drug Administration (FDA) cleared CellSearch test [11,12,13,14,15,16,17]. In addition to the number of CTCs found, their molecular characterization may be a part of a more comprehensive approach providing further information on e.g., downregulation of epithelial markers or presence of druggable targets. Recently, we have demonstrated that processing blood samples using the microfluidic Parsortix™ technology considerably improved the molecular analysis of the enriched CTCs [18].

Considering the abundance of CTCs and the ease of obtaining/performing liquid biopsies extends the possibilities for differential diagnosis and patient stratification. For these reasons we believe that the molecular characterization of CTCs in SCLC may be of uppermost importance for this type of lung cancer. In the present study we applied a recently developed workflow which combines a microfluidic enrichment of CTCs and a qPCR-based analysis for evaluating the gene expression levels of markers of the epithelial (epithelial cell adhesion molecule 1, *EpCAM* and cytokeratin 19, *CK19*) and neuroendocrine (*CHGA*, *SYP*, *NCAM1* and enolase 2 *ENO2*) cell lineage origin, in addition to the druggable target *DLL3*.

## 2. Materials and Methods

Blood samples were taken from patients with SCLC at the Department of Respiratory and Critical Care Medicine at Sozialmedizinisches Zentrum Baumgartner Höhe, Vienna, Austria. Control blood samples came from healthy donors without a history of cancer. All donors signed an informed consent. The study was approved by the Ethics Committee of the Medical University of Vienna, Austria (EK366/2003 and EK2266/2018).

The blood was collected in Vacuette EDTA tubes (Greiner Bio-One) and processed on the same day in accordance with a recently published protocol employing the label-free microfluidic Parsortix™ technology (Angle plc., UK) [18]. The key component of the device is a microscope slide sized disposable separation cassette, which contains a series of steps with a precisely defined height. Rare cells (e.g., CTCs) are captured within the separation cassette based on their less deformable nature and usually larger size compared to blood cells. Before separation, the blood was diluted with an equal volume of phosphate buffered saline (PBS) and directly processed using a Parsortix™ technology. In this study a separation cassette with a critical step size of 6.5 µm was used, and the separation was performed at 99 mbar pressure. After the separation was completed the captured cells were recovered using a back-flush cycle and immediately lysed by adding 350 µl RLT lysis buffer (Qiagen). The lysates were stored at −80 ° C until RNA extraction.

Total RNA was extracted from the cell lysates using the RNeasy Micro Kit (Qiagen) without DNase treatment. The total amount of RNA was converted into cDNA using the SuperScript VILO Mastermix (Invitrogen). qPCR was done in duplicates in a 10 µL total reaction volume on the ViiA7 Real-Time PCR System using the TaqMan^®^ Universal Mastermix II and exon spanning TaqMan^®^ assays specific for *EpCAM*, *NCAM1*, *CHGA*, *SYP*, *DLL3*, *ENO2*, and *CDKN1B* (Life Technologies) with thermal cycling parameters (50 °C for 2 min; 95 °C for 10 min followed by 40 cycles at 95 °C for 15 s and 60 °C for 1 min). A qPCR specific for *CK19* was performed at 65 °C annealing/extension with forward and reverse primers that correspond to published primer sequences and with a FAM™ labeled hydrolysis probe (5’-TgTCCTgCAgATCgACAACgCCC-3’) [19]. Raw data were analyzed using the ViiA7 Software v1.1 with automatic threshold setting and baseline correction. If the fluorescent signal did not reach the threshold in both duplicate reactions, the sample was regarded as negative. 

The SCLC CTC lines used for the spiking experiments were derived from patients’ blood samples [20]. They were trypsinized at about 70% confluence and stained with CellTrace Violet (Invitrogen) according to the manufacturer’s protocol. Subsequently, 100 stained cells were added manually to a 10 mL control blood sample, which was then processed using the Parsortix™ technology as described above. The tumor cells captured in the separation cassette were counted using a fluorescence microscope (Olympus BX50).

The Pearson’s chi-square and Fisher’s exact test were used to assess the relationship between clinicopathological characteristics of the patients and the presence or absence of the respective gene transcripts. Overall survival (OS) was defined as the period of time in months between blood draw and either death or the last date the patient was seen alive. Kaplan–Meier survival analyses and log-rank testing were used to compare survival outcomes [21]. Cox proportional-hazards regression was used to determine univariate and multiple hazards ratios (HR) for OS [7]. The included covariates were the stage of disease at blood draw (primary vs. progressive disease) and the presence vs. absence of the respective transcripts. The model was built using a forward stepwise method by entering all variables at a *p* value of less than 0.05 and removing them at a *p* value of greater than 0.10. The statistical analysis was performed with SPSS version 19.0 (SPSS Inc., Chicago, IL). The level of significance was set at *p* < 0.05. Graphs were done using GraphPad Prism version 6.01. 

## 3. Results

### 3.1. Patients and Samples

The characteristics of 48 patients with a histopathologically confirmed diagnosis of SCLC are shown in Table 1. The SCLC patients were 51 to 78 years old (mean/median age at 64.6/63.5 years), and all patients but one were former or current smokers, with a median of 60 pack years (range 20 to 150). Thirty-four patients died within the observation period, with a median overall survival of 7 months (range 0 to 14 months). The 14 patients who were still alive at study completion were surveyed over a median period of 14 months (range 0 to 19 months). All blood samples were taken before treatment, either at primary diagnosis (*n* = 27), or when progression or recurrence of the disease was observed (*n* = 24). In total, 51 blood samples were available, as blood samples from three patients with progressive disease were taken at two serial time points. The volume of blood was 18 mL in 58.8% of the samples, 17 mL to 14 mL in 33.3%, and 10 mL to 8 mL in 7.8% of the samples. In the control group, 18 mL of blood was taken from 26 healthy donors.

### 3.2. Spiking Experiments

**Figure 1:**
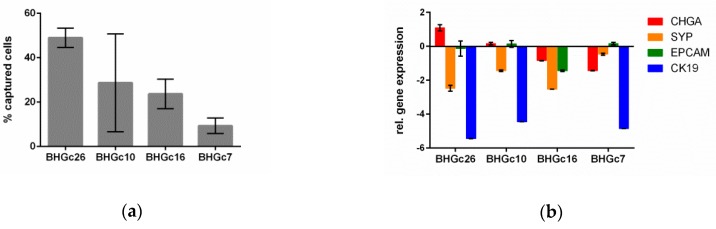
Characteristics of the microfluidic enrichment procedure and of the tumor cell lines used for the spiking experiments are illustrated. (**a**) Four SCLC tumor cell lines (BHGc26, BHGc10, BHGc16, and BHGc7) were fluorescently labeled and spiked into blood (100 cells per 10 mL) in triplicates. The graph depicts the mean percentage and the standard deviation of tumor cells captured in the Parsortix™ microfluidic cassette. (**b**) The gene expression levels of the epithelial and neuroendocrine cell lineage specific markers of the same cell lines are shown relative to the expression level of *cyclin dependent kinase inhibitor 1B* as reference gene. The graphs depict the means and the standard deviation from duplicate qPCRs amplifications.

The efficiency of the microfluidic Parsortix™ system for capturing cultivated SCLC cells derived from four CTC lines [20] in a separation cassette with a critical gap size of 6.5 µm is shown in Figure 1. The overall mean capture efficiency of all four cell lines was 27.8% (SD 16.4%). The gene expression levels of the epithelial (*EpCAM* and *CK19*) and neuroendocrine (*CHGA* and *SYP*) cell lineage origins were assessed in the same four CTC lines using qPCR, showing a wide-ranging pattern of gene expression.

### 3.3. Epithelial and Neuroendocrine Markers in Controls and SCLC Blood Samples

**Figure 2:**
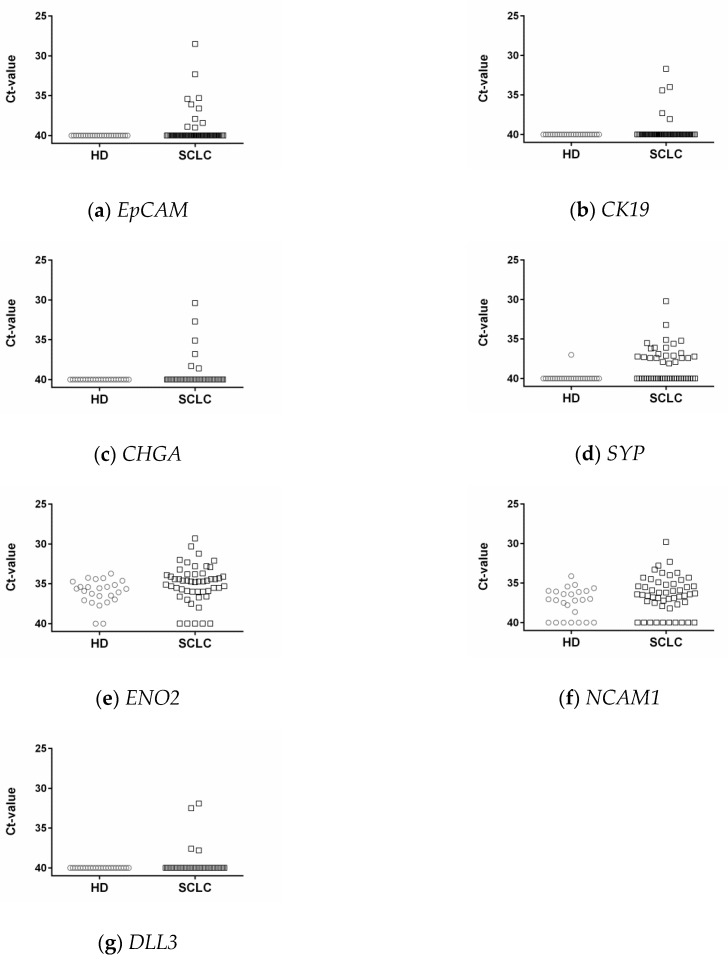
Mean cycle threshold (Ct-) values of the respective transcripts in blood samples taken from 26 healthy donors (HD) and 48 patients with small-cell lung cancer (SCLC). (**a**) *EpCAM*, epithelial cell adhesion molecule; (**b**) *CK19*, cytokeratin 19; (**c**) *CHGA*, chromogranin A; (**d**) *SYP*, synaptophysin; (**e**) *ENO2*, enolase 2; (**f**) *NCAM1*, neural cell adhesion molecule 1; (**g**) *DLL3*, Notch pathway delta-like 3 ligand.

*EpCAM*, *CK19*, and *CHGA* transcripts were not detected in any of the control blood samples (Figure 2a–c). In contrast, *SYP* levels above the detection limit of qPCR were observed in 1/26 (3.8%), and *ENO2* and *NCAM1* transcripts in 24/26 (92.3%) and 19/26 (73.1%) controls, respectively (Figure 2d–f). Due to the high number of *ENO2*- and *NCAM1*-positive healthy donor samples, these markers were considered as less appropriate for CTC detection and thus excluded from further analyses.

In contrast, *EpCAM*-, *CK19*-, or *CHGA*-positivity above the detection limit of qPCR was observed in 10 (19.6%), 4 (7.8%), and 6 (11.8%) of the 51 samples obtained from SCLC patients. Due to the observed *SYP* gene expression in a single control blood sample, the threshold for *SYP*-positivity in the patients’ samples was set at Ct = 37.0. Thus, *SYP* transcript levels below that threshold were observed in 40 (78.4%), and above that threshold in 11 (21.6%) of the 51 SCLC samples. These 11 samples were assigned as *SYP*-positive. In none of the gene transcripts did (*EpCAM*, *CK19*, *CHGA*, and *SYP*)-positivity differ significantly between the blood samples taken at diagnosis and disease progression.

**Figure 3:**
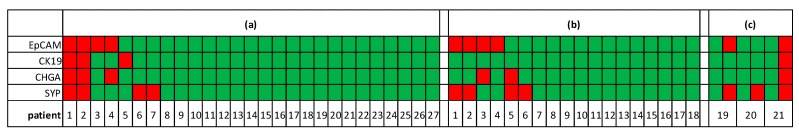
Heat map for *EpCAM*, *CK19*, *CHGA*, and *SYP* in the 51 microfluidic enriched blood samples of patients with small-cell lung cancer. (**a**) Twenty-seven samples were taken at diagnosis, (**b**) 18 samples were taken at progression/recurrence, and (**c**) displays serial blood draws taken from three patients during disease progression. Red and green squares indicate positive and negative gene expression per tested sample, respectively.

In total, 16/51 (31.4%) samples were CTC-positive due to the expression of at least one of *EpCAM*, *CK19*, *CHGA*, and *SYP* markers (Figure 3). The expression of epithelial markers (*EpCAM* and/or *CK19*) was observed in 11 (21.6%), and of neuroendocrine markers (*CHGA* and/or *SYP*) in 13 (25.5%) samples. Among the 16 CTC-positive blood samples, three (18.8%) and five (31.3%) were characterized by the presence of just epithelial or neuroendocrine markers, respectively, and eight samples (50.0%) by both types.

### 3.4. Alterations of Transcript Levels during Disease Progression

From three patients with progressive disease two serial blood samples were taken at the start of the consecutive lines of treatment. In two cases the second blood was taken two months after the first blood draw (patients 19 and 20 in Figure 3c), and in one case after three months (patient 21 in Figure 3c). At the first blood draw all patients were negative in all markers tested; however, at the second blood draw all patients were PCR-positive for at least *SYP* (see Figure 3c). All patients died within 1.5 months of the second blood draw.

### 3.5. Epithelial and Neuroendocrine Specific Markers and Patient Outcome

**Figure 4:**
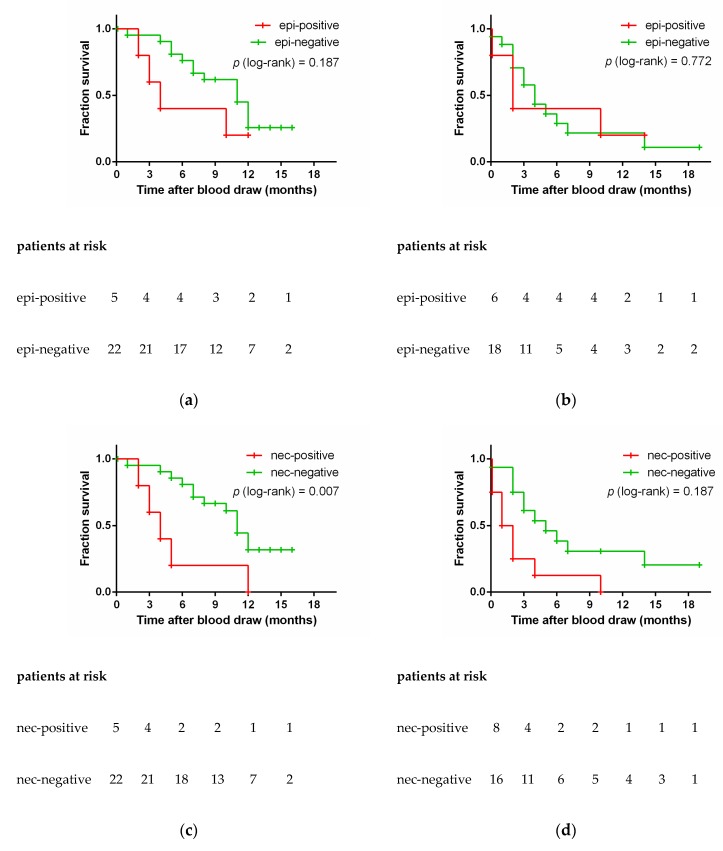
Overall survival of small-cell lung cancer patients according to presence (red) or absence (green) of epithelial (*EpCAM*, *CK19*) and neuroendocrine markers (*SYP*, *CHGA*). The figures (**a**) and (**c**) display samples taken at primary diagnosis, whereas the figures (**b**) and (**d**) display samples taken at progression. Log-rank testing was used to compare survival outcomes. epi, epithelial; nec, neuroendocrine.

The blood samples were stratified on the basis of the epithelial cell lineage-specific gene transcripts *EpCAM* and *CK19* into the epi-positive (*n* = 11) and the epi-negative group (*n* = 40), and on the basis of neuroendocrine-specific transcripts *SYP* and *CHGA* into the nec-positive (*n* = 13) and nec-negative (*n* = 38) group. The presence of *EpCAM* and/or *CK19* transcripts in the epi-positive group at primary diagnosis may be associated with a shorter OS of the patients (Figure 4a); future studies with larger sample sizes may prove whether or not this difference is statistically significant. Similarly, the presence of *EpCAM* and/or *CK19* transcripts at disease progression was not related to OS (Figure 4b). In contrast, nec-positive patients had a significantly shorter OS than nec-negative patients, both at primary diagnosis and at disease progression. That association of *SYP* and/or *CHGA* transcripts with OS was observed with the presence of these neuroendocrine markers both at primary diagnosis (median OS 4 vs. 11 months, log-rank *p* = 0.007; Figure 4c), as well as at progression (median OS 1 vs. 5 months, log-rank *p* = 0.014; Figure 4d). Irrespective of whether the sample was taken at primary diagnosis or at disease progression, nec-positive patients had a high-risk of an early death (HR 3.475, 95% CI 1.685–7.164; *p* = 0.001).

### 3.6. DLL3 in Controls and SCLC Blood Samples

**Figure 5:**
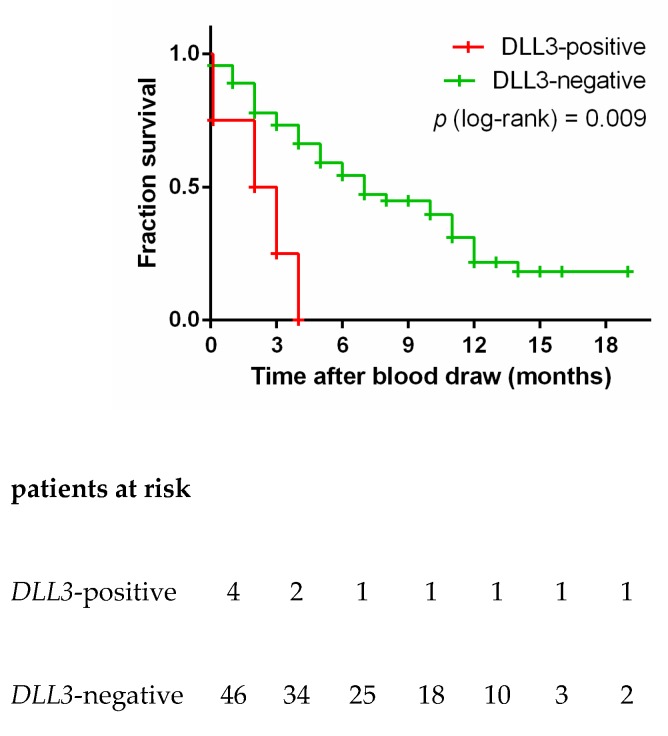
Overall survival according to presence (red) or absence (green) of *DLL3*. Log-rank testing was used to compare survival outcomes.

*DLL3* transcripts were observed in 4/51 (7.8%) of the SCLC blood samples and in none of the 26 control blood samples (Figure 2g). Three *DLL3*-positive blood samples were taken at primary diagnosis, and one was taken from patient 19 at the second blood draw (see Figure 3c). Due to the small number of DLL3-positive patients, we did not stratify the patients into two groups depending on the time-point of blood draw. All four *DLL3*-positive patients had a significantly shorter OS than *DLL3*-negative patients (median OS 2 vs. 7 months, log-rank *p* = 0.003; Figure 5). The risk of dying earlier was 3.793 (95% CI 2.803–115.6) higher in the *DLL3*-positive group compared to the *DLL3*-negative group.

## 4. Discussion

We have applied a recently established workflow for molecular detection of CTCs [18] in blood samples taken from patients with SCLC, which is a highly aggressive neuroendocrine tumor of the lung. The enrichment of the CTCs was achieved with the microfluidic Parsortix™ technology, and the molecular analysis of the harvested cells was performed using markers that are specific to epithelial (*EpCAM* and *CK19*) and to neuroendocrine cell lineages (*SYP*, *CHGA*, *ENO2*, *NCAM1*), and to *DLL3*, an actionable target of antibody-drug conjugate rovalpituzumab tesirine (Rova-T). To the best of our knowledge, this is the first study that investigates neuroendocrine markers and *DLL3* in CTCs of SCLC patients at a molecular level.

We detected *EpCAM* and/or *CK19* transcripts in 21.6%, and neuroendocrine *CHGA* and/or *SYP* transcripts in 25.5% of the 51 SCLC blood samples. Interestingly, five of the 16 (31.3%) qPCR-positive samples were identified by the presence of neuroendocrine-specific transcripts alone. Similarly, three (18.8%) CTC-positive samples expressed the epithelial markers alone.

The percentage of CTC-positive samples due to the expression of epithelial markers of 21.6% in our cohort is smaller than reported by others in SCLC [22]. Applying the FDA approved CellSearch-based approach for the detection and enumeration of CTCs, positive findings were observed in 50% to 86% of the patients by other investigators [12,23,24]. The reason for the low detection rate of CTCs in our study may be the low overall sensitivity of our approach reflecting the need to split the sample into aliquots to analyze the expression of multiple genes individually. Improved sensitivity could be achieved by multi-plexing the gene expression analysis to avoid splitting the sample. A further improvement of the overall approach may also be achieved by employing a gene-specific pre-amplification of the respective targets prior to qPCR. In a recent study we have demonstrated that targeted pre-amplification in Parsortix™-enriched blood samples is feasible [18].

Another clear limitation of our study is the possibly low efficiency of the enrichment procedure to isolate CTCs from SCLC blood samples. The spiking experiments showed only moderate capture rates of SCLC CTC lines (mean capture efficiency 27.8%, SD 16.4%). These capture rates are lower than reported for the breast cancer cell line MCF-7 using the same type of separation cassette (average 63% [25]). In line with our observations, the capture rates varied depending on the type of cell line used from 30% to 87% in renal carcinoma [26]. The four cell lines used in the present study had been established from patients’ CTCs [20]. Their diverse gene expression pattern (see Figure 1b) may reflect the initial heterogeneity of their provenance and might contribute to varying capture efficiencies. In the present study we did not check the number of tumor cells after harvesting; however, results from a recent study imply that the recovery rate may vary depending on the type of cell line from 62%–84% [27]. The number of harvested tumor cells can be increased by intensifying the back-flush cycle; however a higher recovery will only be achieved at the cost of a lower purity of the tumor cells.

Using larger volumes of blood may be a further attempt to increase the sensitivity of the assay in future studies. In our study all four samples with a volume of 10 mL blood or less were negative for all gene markers investigated. We did not exclude these few blood samples from the survival analyses shown in Figure 4 and Figure 5, as that would not alter the significance of the analyses.

There is a single study applying the Parsortix™ technology for the enrichment of blood samples from SCLC patients. In that study, Chudziak et al. found CTCs in all 12 patients, as assessed by immune-fluorescent cytokeratin-specific staining of the enriched cells [16]. In contrast to our approach that group used blood collection tubes containing a preservative which is known to increase the rigidity of the cell, and thereby increasing the number of cells captured in the microfluidic cassette. This fact, along with the more advanced stage of disease in their study population may be the reason for the divergent CTC-positivity rate obtained in that study as compared to ours.

Another weakness of our study may be the limited sample size. Because of that and the low overall sensitivity we had very few positive samples. Thus we were not able to investigate association of the patients’ prognosis and the respective gene expression levels, and the interplay of epithelial and neuroendocrine markers in a more detailed way.

High numbers of CTCs at diagnosis, as assessed by CellSearch, were associated with a poor prognosis (reviewed by [10]), yet the investigators reported divergent CTC numbers as a threshold for defining a group of patients with poor prognosis. [14,23]. However, we did not observe any significant impact of the expression of the epithelial markers on the OS. Nonetheless, patients who were epi-positive at primary diagnosis died earlier than epi-negative patients. A statistical significance may be reached by increasing the number of patients in future studies.

Studies investigating CTCs in other neuroendocrine tumors, such as those originating from the prostate, thyroid gland, or the intestine, mainly applied epithelial cell lineage-specific markers and protein-based technologies for the enrichment and analysis of CTCs [28]. However, CTCs may be missed when epithelial markers, such as *EpCAM*, are downregulated in the tumor tissue, as was shown in neuroendocrine tumors of the lung [29]. In addition, tumor cells can undergo epithelial-to-mesenchymal transition and lose their epithelial phenotype [24]. In the present study we detected *CHGA* and/or *SYP* transcripts in 13 samples; this absolute number corresponds to 25.5% of all 51 SCLC blood samples, and to 81.3% of all 16 CTC-positive samples. That the percentage is still not 100% may be because of low numbers of CTCs in some samples. Furthermore, Guinee et al. demonstrated the absence of neuroendocrine markers in just about 20% of the specimen by immunohistochemical staining [6]. The fact that one third of the qPCR-positive samples was identified by the presence of these neuroendocrine transcripts already indicates that epithelial markers alone may not be sufficient to detect CTCs in neuroendocrine tumors such as SCLC. One observation in this respect is of particular interest. The presence of selected neuroendocrine markers was associated with a worse outcome and not the presence of used epithelial markers. This even applies irrespective of the time the markers were detected—be it prior to treatment at initial diagnosis or when the disease has already progressed.

To the best of our knowledge there is just a single study investigating the clinical relevance of neuroendocrine markers in CTCs: Recently, Pal and colleagues quantified the percentage of *SYP*-positive CTCs in blood samples taken from castration-resistant prostate cancer patients using the open fluorescent channel of the CellSearch platform [30]. They observed an increasing number of *SYP*-positive CTCs with the onset of resistance to androgen-receptor targeting drugs, which are assumed to stimulate the transition to the neuroendocrine phenotype [31].

## 5. Conclusions

Besides the neuroendocrine markers *SYP* and *CHGA*, our study also clearly shows that *DLL3* can be detected in CTC-enriched blood samples. Traditional patient stratification for personalized treatment options, such as Rova-T, is based the analysis of tissue samples that were taken long before the disease progression occurred. In contrast, liquid biopsy samples can be taken right before the start of treatment, and may thus provide a snapshot analysis of promising targets for personalized treatments, such as *DLL3*. Apart from treatment stratification, liquid biopsies can be taken at several consecutive points in time to assess the response to treatment. Despite the promising results of our study, the findings need to be validated in larger studies of SCLC patients. In conclusion, the molecular analysis of CTCs may add relevant information to traditional tissue biopsies or cytological specimens in small-cell lung cancer patients, especially in treatment selection and patient monitoring.

## Figures and Tables

**Table 1:** Characteristics of 48 small-cell lung cancer (SCLC) patients included in the study.

Characteristics	*n* (%)
Age Mean (median) Range	63.5 y (64.6 y) 51.0–78.0 y
Gender Male Female	30 (62.5%) 18 (37.5%)
Tobacco abuse Current smokers Former smokers Never smokers Unknown	13 (27.1%) 26 (54.2%) 1 (2.1%) 8 (16.7%)
UICC 8th edition TNM stage at diagnosis^1^ III IV Unknown	4 (11.4%) 31 (88.6%) 13 (27.1%)
Outcome at study completion Dead Alive	34 (70.8%) 14 (29.2%)
Blood draw for CTCs At primary diagnosis At progression/recurrence	27 (56.3%) 21 (43.8%)

^1^ UICC, International Union for Cancer Control.
